# A Comparative Study on Willingness to Pay for Breast Cancer and Osteoporosis Screening in Kerman, Southeastern Iran

**Published:** 2017-05

**Authors:** Asma SABERMAHANI, Sedighe MOHAMMAD TAGHIZADE, Reza GOODARZI

**Affiliations:** 1. Health Services Management Research Center, Institute for Futures Studies in Health, Kerman University of Medical Sciences, Kerman, Iran; 2. Iran Health Insurance Organization, Kerman, Iran; 3. Modelling in Health Research Center, Institute for Futures Studies in Health, Kerman University of Medical Sciences, Kerman, Iran

**Keywords:** Willingness to pay (WTP), Screening, Osteoporosis, Breast cancer

## Abstract

**Background:**

One of the economic evaluation techniques involves calculation of willingness to pay (WTP) for a service to find out the value of that service from the clients’ perspective. This study estimated WTP for both breast cancer and osteoporosis screening and comparatively examined the contributing factors. In fact, the comparisons served to provide an exact analysis of individual attitudes and behaviors in relation to screening programs for cancers and other diseases.

**Methods:**

This study was first designed in six scenarios several questionnaires concerning individual breast cancer and osteoporosis screening cases, and determined the WTP median in each scenario between people in Kerman Province of Iran in 2016. Then, the demand function for breast cancer and osteoporosis screening was formulated. Moreover, the factors contributing to WTP were examined through various scenarios in Stata and econometric techniques.

**Results:**

The median and mean values of WTP in all the above scenarios were greater for breast cancer screening than for osteoporosis screening. Theoretically, the price assumed a minus sign whereas risk assumed a plus sign within the demand function formulated for both screening programs. Regarding the evaluated factors, age in breast cancer screening and risk of disease in osteoporosis screening were the major factors contributing to WTP.

**Conclusion:**

Breast cancer screening was more valuable than osteoporosis screening program from the perspective of the subjects. The programs can be successfully designed by concentrating on patients’ age groups in breast cancer screening and high-risk patients in osteoporosis screening.

## Introduction

Since about 80% of deaths due to noncommunicable diseases occur in low-and middle-income countries and the most frequent causes of death in most countries, except in Africa, are NCDs (1), early prevention and diagnosis of these diseases are high on the agenda of the majority of these countries. Similarly, in the Iranian health care system, great attention has been focused on screening, particularly for cancers. In this regard, several plans have been implemented in recent years. One of these pilot plans was breast cancer screening in Kerman Province, Iran, to survey about cost effectiveness and people contribution in the program. Results of breast cancer screening program showed that it is not cost effectiveness because of very low contribution of population. After that, osteoporosis screening was planning in health insurance organization of Kerman, so having information about economic evaluation of the plan was very useful.

It is a difficult task to measure the benefits of prevention or health promotion programs. Calculation of willingness to pay (WTP) can provide a technique to overcome that difficulty based on the principles of welfare economics. The WTP has been applied in studies where the decision-makers were government organizations and the stakeholders the society as a whole (2) and osteoporosis-screening plan was one of them.

This study focused on WTP for breast cancer screening among women living in Kerman, Iran. This WTP was compared with the corresponding WTP obtained for osteoporosis screening with regard to numerical values and the contributing factors. Such comparison mainly served to provide an exact analysis of individual attitudes and behaviors toward the value of screening programs (3–5) in cancers and other diseases to find out whether or not fear of being diagnosed with cancer led to lower values of screening programs compared to the non-cancerous diseases. In other words, is it possible that people more contribute in osteoporosis screening than breast cancer screening program? If WTP for osteoporosis screening was higher than breast cancer screening so, we can expect more contribution in the plan. Another objective of this study was to compare the most important factors contributing to WTP in the two screening programs.

Many researches use this technique in health economic evaluations in other countries (6–8) but in Iran, researchers can find one similar article (9) in health sector. In that article, WTP for mammographic breast cancer screening in Tehran was calculated in 2010. We conducted our research in different base for giving more detailed and suitable results for a comparative study.

## Material and Methods

This was an applied, descriptive-analytical study involving field data collected from four culturally and economically different regions in Kerman, Iran during 2015. The regions were selected through expert opinion as contain all parts of city. Samples were gathered in proportion to the population of each region randomly. In the first stage, the scenarios were designed as a pilot through open-ended questions aiming to examine the WTP for breast cancer and osteoporosis screening. The scenarios were described to each subject who offered reasonable prices. Then, a mean value was obtained as a starting point for the proposed prices within the questionnaire. Each questionnaire consisted of six scenarios designed in two sections.

In three scenarios, people were asked to assume high risk of disease for themselves and in three scenarios assume low risk of disease and answer the questions. Each section, scenarios were about individual WTP for implementation of a plan for screening all people with increasing government contribution in tree level (tree scenarios) 10, 50 and 90 percentages of the plan expenditures. These tree levels were designed to check reasonability of people answers. Declared prices increase with increase in level of government contribution. The first section of the questionnaire provided explanations about the disease, followed by several items revolving around WTP. Finally, there were a number of items to estimate the income, age, and gender of subjects (related to osteoporosis). As recommended by the economic studies, the subjects were asked about their monthly expenses instead of their income. Several experts in economics of health verified the WTP questionnaires. About 50 questionnaires in each category of breast cancer and osteoporosis were examined in another pilot to ensure the responses entail homogeneity and make sense.

In WTP studies, the number of samples can be calculated according to Mitchell and Carson’s method, who designed a table displaying the minimum required sample size for acceptable levels of reliability and error in contingent valuation method (CVM) studies (10). This table was used to determine a set a sample size of 458 for each WTP study (breast cancer and osteoporosis screening) based on a relative error of 1.5 and a reliability level of 0.05. For greater accuracy, a sample size of n=600 was adopted. The sample size covered women of the 35–69 yr ages group for breast cancer screening and women/men over 55 yr for osteoporosis screening.

Having designed the questionnaire at the training stage, we held several in-person interviews in each studied region, trained the health volunteers and delivered the questionnaires to them. After a twenty-day deadline, the questionnaires were completed, and their data subsequently collected and analyzed. At this stage, the data were analyzed to ensure none was corrupted.

The WTP in each scenario was obtained by suggesting prices that were higher and lower than the initial price the subjects had, depending on their preferences, either accepted or rejected. Finally, the last choices made by the subjects were collected to obtain the individual WTP in each price scenario. Although it is better to use the median rather than the mean in calculation of WTP, the latter was also considered in order to draw more extensive comparisons of WTP between the two screening programs.

After calculating the cumulative frequency at different prices, the demand function was formulated by inserting the risk factors and integrating the first and fourth, second and fifth, and third and sixth scenarios, which were different only in terms of risk levels.

Then, the regression analysis was employed to identify the factors contributing to WTP. This stage explored the risk factors, age, and income. As for the osteoporosis screening, the impact of gender was examined by importing a few dummy variables. Furthermore, the data were analyzed in terms of variance heterogeneity, collinearity, and other econometric pre-tests.

## Results

The median of WTP in all the proposed scenarios tended to be higher for breast cancer screening than for osteoporosis screening (Table 1).

**Table 1:** Median of WTP for breast cancer and osteoporosis screening

**Scenario**	**1**	**2**	**3**	**4**	**5**	**6**
Median of WTP for breast cancer screening	20	20	180	10	10	125
Median of WTP for osteoporosis screening	10	10	150	5	5	90

The mean of WTP in various scenarios was higher for breast cancer screening. The results are presented in Table 2. The data distribution was examined by the Shapiro-Wilk test, where the null hypothesis of normal data distribution was rejected at confidence level of 95%. Since the data distribution was not normal, the Mann-Whitney U test was employed to prove the significance difference between the mean values. The results of Mann-Whitney test can be seen in Table 3.

**Table 2:** Mean of WTP for breast cancer and osteoporosis screening

**Scenario**	**1**	**2**	**3**	**4**	**5**	**6**
Mean of WTP for breast cancer screening	31.5	32.6	190.2	20.6	21.2	131.1
Mean of WTP for osteoporosis screening	22.7	23.8	153.2	15.4	15.8	102.8

**Table 3:** Significant difference of the mean WTP in the first six scenarios between breast cancer screening and osteoporosis screening

	**S1**	**S2**	**S3**	**S4**	**S5**	**S6**
Z	4.65	3.26	5.54	5.01	3.7	6.99
Prob > |z|	0.000	0.001	0.000	0.000	0.000	0.000

The results of demand function formulation for breast cancer screening and osteoporosis screening are given in Tables 4 and 5 respectively. Theoretically, price (lp) and demand (lq) were inversely correlated with both demand functions, whereas there was a positive relationship between risk of disease and demand.

**Table 4:** Demand for breast cancer and osteoporosis screening

**C**	**Breast Cancer**			**Osteoporosis**		
	**Coefficient**	***t***	***P*>*t***	**Coefficient**	***t***	***P*>*t***
lp	0.52-	−10.92	0.000	−0.58	−10.61	0.000
Risk	2.5	16.38	0.000	0.42	2.28	0.026
_cons	5.8	24.62	0.000	7.99	31.7	0.000

**Table 5:** The factors contributing to WTP for breast cancer screening

**Lwtp**	**Coefficient**	**t**	***P*>|t|**
Risk	0.37	5.10	0.000
Lage	−0.55	−3.02	0.003
Expenditure	0.27	7.34	0.000
Constant	6.08	8.70	0.000

In the formulated demand functions, the Breusch–Pagan test confirmed the heterogeneity of variance, whereas the VIF results confirmed there was no co-linearity.

Table 5 illustrates the factors contributing to WTP for breast cancer screening. Risks, household expenses/income had a significantly positive impact on WTP, whereas the age was in a significantly inverse correlation with WTP.

Table 6 illustrates the factors contributing to WTP for osteoporosis screening. The regression estimates showed that greater risk and income led to a significant increase in WTP. Furthermore, increasing age had a positive but statistically insignificant effect on willingness. As for the effect of gender, men are more willing to pay than women are, even though such an effect was not statistically significant.

**Table 6:** The factors contributing to WTP for osteoporosis screening

**Lwtp**	**Coefficient**	***t***	***P*>*t***
Risk	0.45	6.38	0.000
Lage	0.37	1.37	0.171
sex	0.07	1.01	0.311
Expenditure	0.11	3.01	0.003
constant	2.46	2.21	0.027

## Discussion

The median of WTP or the value of the program from individual perspectives in all scenarios was greater for breast cancer screening than osteoporosis screening. Moreover, the mean difference of WTP for the two screening programs confirmed that WTP in all scenarios was greater for breast cancer screening. In this regard, the difference was statistically significant. This finding was inconsistent with the assumption that subjects tended to perform osteoporosis screening rather than breast cancer screening due to lower fear of being diagnosed in non-cancerous cases. In fact, individuals believe breast cancer screening is more valuable. This finding can be justified through the importance of cancers, the critical aspect of early diagnosis and the peace of mind achieved through cancer screening (11, 12). People contribution in osteoporosis screening program is lower than breast cancer screening and it means lower cost effectiveness.

Assuming an increase in the risk of disease for subjects in both screening programs, there was greater WTP. This was consistent with all the studies focusing on the impact of risk on WTP (13).

According to the results, there was no significant difference between women’s and men’s WTP for osteoporosis screening. Although women tended to be more risk-averse than men were. Women and men are equally risk-averse under conditions where uncertainty intensifies (14). Since the risk of disease and the need for screening services are quite uncertain, equal WTP makes sense.

The price left a negative effect on demand and a positive effect on risk. Theoretically, there is an inverse correlation between price and demand (15), indicated by a minus sign within the function. The impact of price increase on reducing the demand was greater in osteoporosis screening. Since the coefficients of the demand function indicate price elasticity and the price coefficient is greater for osteoporosis, the price elasticity for osteoporosis screening was estimated to be greater than breast cancer screening. Individuals were less concerned about the price of breast cancer screening.

In examining the factors contributing to WTP for breast cancer and osteoporosis screening, risk or household expenses/income had a positive effect on WTP, and that this relationship was identical in both screening programs. Individuals with greater risk and income were more willing to pay. Moreover, patient’s age was inversely correlated with WTP for breast cancer screening, whereas it was directly correlated for osteoporosis screening. Older people believe to be at a greater risk for osteoporosis. Consequently, older individuals were more likely to perform osteoporosis screening. On the other hand, the risk of breast cancer increases with age (16) even though breast cancer screening was less valuable to older women, perhaps because they believed to be more immune to breast cancer than younger women. That some women think screening is an unnecessary measure reflects poor social awareness with regard to medical facts.

WTP was affected significantly and positively by household income (questioned under “household expenditures” in the questionnaire to reduce bias). The positive impact of income on WTP was proven (9).

This study had some limitations that one of them is no direct supervision on filling questioners. It was not possible because of large sample size. For reduction this limitation, researchers held several training classes for the health volunteers. However, in the regression like other regressions, some factors were not considered, too. Doing open interviews about factors, affecting willingness to pay could complete the research that was not possible for the researchers so they had not entered some qualitative factors in the model.

## Ethical considerations

Ethical issues (Including plagiarism, informed consent, misconduct, data fabrication and/or falsification, double publication and/or submission, redundancy, etc.) have been completely observed by the authors.
